# Impact of Synchronous and Asynchronous Settings of Online Teaching and Learning in Higher Education on Students’ Learning Experience During COVID-19

**DOI:** 10.3389/fpsyg.2021.733554

**Published:** 2021-10-11

**Authors:** Sabine Fabriz, Julia Mendzheritskaya, Sebastian Stehle

**Affiliations:** Department of Educational Psychology, Institute of Psychology, Goethe University, Frankfurt, Germany

**Keywords:** higher education, self-determination theory (SDT), COVID-19, teaching and learning settings, online learning

## Abstract

The sudden impact of the COVID-19 pandemic challenged universities to provide students with online teaching and learning settings that were both immediately applicable and supportive of quality learning. This resulted in a broad variety of synchronous and asynchronous online settings of teaching and learning. While some courses balanced both kinds, others offered either predominantly synchronous or asynchronous teaching and learning. In a survey study with students (*N*=3,056) and teachers (*N*=396) from a large German university, we explored whether a predominance of synchronous or asynchronous teaching and learning settings in higher education was associated with certain student experiences and outcomes. Additionally, we examined how well these two types of teaching and learning settings support students’ basic psychological needs for autonomy, competence, and relatedness proposed by self-determination theory (SDT). Data were collected after the first online semester due to the COVID-19 pandemic. The results imply that from the students’ perspective, the teaching methods involved in the two settings of teaching and learning differ with regard to their potential to support social interaction and to support basic psychological needs as proposed by SDT. Students who studied mostly in synchronous settings reported more peer-centered activities such as feedback in comparison to students in mostly asynchronous settings. In contrast, teachers perceived fewer differences between teaching methods in synchronous and asynchronous settings, especially regarding feedback activities. Further, students in mostly synchronous settings reported greater support of their basic psychological needs for competence support and relatedness as well as a greater overall satisfaction with the online term compared to students in mostly asynchronous settings. Across all students, greater fulfillment of psychological needs and higher technology acceptance coincided with outcomes that are more favorable. Implications for the post-pandemic classroom are drawn.

## Introduction

The sudden need to adapt to online teaching and learning due to the COVID-19 pandemic challenged the digital readiness of teachers and students all over the world (Bao, 2020; Crawford et al., 2020; Demuyakor, 2020; Händel et al., 2020; International Association of Universities, 2020). The result, called *emergency remote teaching* (ERT, Hodges et al., 2020), included a great amount of improvisation and *ad hoc* strategies that need to be contrasted to well-planned online learning scenarios (e.g., Rapanta et al., 2020). The initial emergency state has since transitioned into the *post-pandemic* or *post-COVID classroom* (Curtin, 2021), in which higher education institutions have the opportunity to integrate those remote teaching practices which have proven their worth into thoroughly planned online or blended learning arrangements while refining or omitting ineffective practices.

In ERT, almost all face-to-face teaching was substituted through online teaching formats (Zawacki-Richter, 2020; Cicha et al., 2021; Goertz and Hense, 2021). This transition was accompanied by the awareness that the pedagogy needed to be adapted to the new medium in the sense that simply moving pedagogy from one medium into another was not enough to ensure quality learning (Henriksen et al., 2020). In addition, students and teachers not only needed new skills in handling technology but also in interacting with each other, resulting in newly shaped roles (Coppola et al., 2002; Arbaugh, 2004; Granitz and Koernig, 2011; Blumentritt et al., 2020). During the pandemic, the social aspect of university learning was especially challenging, resulting in reports of anonymity and a lack of social presence. In a study prior to the pandemic, Daigle and Stuvland (2020) found this lack to account for differences between modalities regarding, for example, lower satisfaction with online learning. They described this as the *social presence gap* and claimed that teachers should invest in overcoming this gap to equalize outcomes across modalities. For many students, the unaccustomed distance in their learning was challenging, for example, Bedenlier et al. (2020) found that students felt uncomfortable using their webcams in synchronous settings. The authors attributed this to the unfamiliar setting, in which they constantly see themselves, and it remains unclear who can see them. Also, students perceived diffuse relationships to their peers and were less likely to experience social support in settings of online learning compared to traditional settings (Bedenlier et al., 2020). In addition, many students also reported an increased workload (Aristovnik et al., 2020). Overall, these findings stress the importance to carefully consider students’ learning experience when tackling the question of how to engage them in online learning.

In online learning, two basic settings are often compared, asynchronous and synchronous. They differ in terms of time and place of teaching and learning activities: Asynchronous settings are temporally and geographically independent and defined as more individually based and self-paced as well as less instructor-dependent (Bernard et al., 2004; Murphy et al., 2011; Clark and Mayer, 2016; Xie et al., 2018). They, however, also bear challenges, as also implied by the media richness (Daft and Lengel, 1984; Blau et al., 2017) and media naturalness (Blau et al., 2017) approaches. The media richness approach describes the “capability of a medium to (1) provide immediate feedback (2) transmit verbal and non-verbal communication cues (3) provide a sense of personalization, and (4) simulate a natural language” (Blau et al., 2017), whereas for the degree of medium naturalness, face to face is considered to be the most natural form of communication. This results in synchronous learning environments to be less natural and less “rich” than face-to-face synchronous learning environments. The authors therefore claim that this leads to higher cognitive load, greater communication ambiguity, and lower activation. And albeit asynchronous teaching can enable students to work self-paced and independently of time and place (van der Keylen et al., 2020), not all learners are equipped with the according strategies to benefit from this potential advantage: Learning at home, especially in asynchronous contexts, requires more self-study skills to stay on track, including enough motivation and will to follow learning goals (*cf*. Hartnett, 2015). Also, students must be equipped with strong digital skills to perform academic work and successfully complete learning activities (Kim et al., 2019).

The main strengths of synchronous online learning are the real-time interpersonal communication, the use of natural language, and immediate feedback (Blau et al., 2017). These attributes can diminish the difference between online and face-to-face learning in this manner and provide a sense of personalization. In contrast, synchronous communication has been found to be less useful for discussing complex ideas or deep reflection (for a review, see Hrastinski, 2010). For students, learning experience, positive outcomes, and the type of performance matter: They acquire practical skills better when they are taught in a synchronous online setting (Nsa et al., 2012; Ogbonna et al., 2019), whereas cognitive achievement, such as producing meaningful and thoughtful contributions, is greater in asynchronous settings (Hrastinski, 2008; Garrison, 2011; Ogbonna et al., 2019). Also, synchronous learning positively impacts learners’ commitment and their task motivation (Hrastinski, 2008). At the same time, similar to face-to-face settings, the danger of disengaged participation in class (e.g., passive listening or watching the teacher’s lecture, silently reading peer statements in the chat) has to be considered (Smith and Smith, 2014). According to an interview study with experts on online teaching by Rapanta et al. (2020), videoconferencing decreases the fluency of interaction and makes interactions slower and attention lower compared to traditional teaching (Rapanta et al., 2020). Another challenge of synchronous learning relates to the technical infrastructure that has to allow for participation in live remote settings in a sufficient quality (i.e., internet bandwidth; Xie et al., 2018).

Research findings regarding the impact of synchronous and asynchronous teaching settings on student performance are not without ambiguity. Nieuwoudt (2020) found that it did not make a difference for student achievement whether students attended synchronous virtual classes or watched the recordings of the virtual classes. However, the sheer time students participated in and interacted with the online learning system did significantly affect their academic success. Also, active participation in both synchronous and asynchronous online learning opportunities has been found to result in higher engagement and better academic outcomes than attending face-to-face classes only (Northey et al., 2015).

In order to scrutinize the impacts of synchronous and asynchronous online teaching and learning on student variables, it is necessary to consider the role of specific teaching methods and the underlying pedagogy of the online courses (Murphy et al., 2011). Synchronous and asynchronous settings differ in the choice of tools used and their pedagogical objectives. Xie et al. (2018) identified five variables to differentiate between synchronous and asynchronous settings: communication tools, feedback types, input methods, collaboration modes, and the skills targeted. The researchers find that while students are more satisfied with asynchronous communication tools (such as discussion forums or email communication), they also appreciate the possibility of direct instructor feedback in synchronous settings. Also, both the quality of learner-content interaction (i.e., reading interactive texts, watching videos, and completing assignments), and learner-teacher interaction (i.e., providing feedback, providing summative and formative assessments, and documenting students’ progress) have a strong effect on satisfaction with learning and perceived learning, especially in asynchronous formats (Kuo et al., 2014; Nandi et al., 2015; Alqurashi, 2019; Fredericksen et al., 2000). Activities, such as online discussions, are perceived as more individualistic and less cooperative by students in asynchronous compared to synchronous settings and are also associated with greater negative effects and a decreased sense of belonging (Peterson et al., 2018). In contrast, learners characterize participation in online synchronous discussions as more focused, having a stronger sense of contribution, increasing motivation, and supporting better course performance than asynchronous discussions (Chen and You, 2007; Hrastinski, 2008, 2010; Malkin et al., 2018). Discussing teaching and learning methods to facilitate communication within synchronous and asynchronous educational settings, researchers stress the necessity to differentiate between various types of activation and interaction and ways how students are engaged in the learning process as more crucial for study success compared to the form of course delivery (Zhu, 2006; Skylar, 2009; Nieuwoudt, 2020; Rapanta et al., 2020; Sweetman, 2021).

Applying criteria for interactivity, teaching and learning methods can be classified in methods with higher versus lower interaction potential. Interactivity in this context refers to the possibility for learners to be socially and cognitively engaged in (1) interaction with content through learning materials, (2) interaction with peers, and (3) interaction with teachers (Anderson, 2003). According to this classification, collaborative formats as discussion, feedback, and working in small groups have higher potential to support social interaction and engagement of students in contrast to lecturing, self-assessments, or individual work which have higher potential for content-oriented interaction in online learning (Rapanta et al., 2020). Similar aspects of student activation and interaction are considered in a well-established classification of student-centered and teacher-centered teaching and learning methods that are usually linked to different degrees of active or correspondently passive learning (Kain, 2003; Chi, 2009; Biggs and Tang, 2011; Wright, 2011) including online learning as well (e.g., Reaburn et al., 2009). A distinguishing parameter of asynchronous versus synchronous online learning is the prevailing learner-content (*via* learning materials) interaction in asynchronous settings in comparison with learner-instructor or learner-learner interaction (Alqurashi, 2019).

Engaging students in online learning is considered a pivotal prerequisite for their success (Chiu, 2021). Also, learners’ motivational characteristics, such as technology acceptance, are often considered factors that can influence achievement or learning satisfaction in synchronous versus asynchronous online courses. The self-confidence in utilizing technologies used in the online course or communicating with a teacher or peers *via* tools is strongly linked to perceived learning and satisfaction (Shen et al., 2013; Alqurashi, 2016; Malik and Fatima, 2017). In general, the facets of technology acceptance – *perceived ease of use* (PEOU) and *perceived usefulness* – are considered significant factors for adopting online teaching and learning environments (see Šumak et al., 2011 for a meta-analysis), irrespective to the type of online resource (e-learning system or single e-learning tool/technology). Recent studies add evidence on the role of technology acceptance in adoption of specific technology-based activities such as online collaboration for problem-based scenarios (Cheung and Vogel, 2013). Very few studies pay attention to the role of technology acceptance in utilizing online learning under the circumstances of the COVID-19 pandemic (i.e., Cicha et al., 2021) showing new patterns of interrelations between technology acceptance, computer anxiety, and self-efficacy.

To investigate prerequisites for learning motivation in synchronous and asynchronous online learning, the self-determination theory (SDT, Ryan and Deci, 2000) presents a befitting framework (Hartnett, 2015; Chiu, 2021). SDT argues that three fundamental psychological needs have to be satisfied for people to act intrinsically motivated in a given environment and to engage with learning: First, people need to feel self-determining or autonomous in their decisions and, through this experience, a sense of control. Second, they need to feel competent or capable to comply with the demands of a given task. Third, they have to feel socially related to or included in a group of others. If a learning context satisfies these basic psychological needs, learners are likely to act intrinsically motivated by, for example, engaging actively in the learning tasks, showing enhanced performance and demonstrating greater endurance when faced with obstacles (Schunk et al., 2014). The key concept for supporting motivation in SDT is the social context. In learning settings, social interactions with the teacher and fellow students can all provide the basic needs of autonomy, competence, and relatedness. One of the benefits of SDT is that it equips teachers with practical advice regarding the kinds of social interactions that students need in order to provide sufficient support for all three basic needs (e.g., granting choice regarding contents or the execution of tasks, offering informational feedback, and assigning group tasks; e.g., Reeve and Jang, 2006). SDT has been successfully applied to classic face-to face-educational settings (Niemiec et al., 2006): Previous studies show that SDT can predict a range of learning outcomes, such as performance, persistence, and course satisfaction (Deci and Ryan, 1985). The social context of online learning differs fundamentally from that of traditional face-to-face learning: Communication takes place through video conferencing tools, forums, chat tools, or email in asynchronous settings, because learners and teachers in online settings of teaching and learning are separated by time, distance, or both. Thus, it seems reasonable to pay special attention to the social context when investigating the link between online learning and teaching settings and learning motivation. For example, previous studies have shown that lack of teacher input, not having a genuine reason to communicate online with peers, low self-efficacy, and time and technology constraints can lower motivation (Xie et al., 2006; Artino, 2007; Cheung et al., 2008; Moos and Azevedo, 2008; Hartnett et al., 2011). By emphasizing the importance of the social context for motivation, SDT is particularly suited to the context of online learning. Some research has previously applied SDT to online learning and learning: A recent study by Chiu (2021) investigated how SDT could explain engagement of students in high school during COVID-19 and found that especially the support of relatedness was important. Also, Hartnett (2015) adopted SDT to an online environment and identified several influences that might undermine the psychological needs: high workload, assessment pressure, perceptions that the learning activity lacked relevance (autonomy-undermining), unclear and complicated guidelines, insufficient guidance and feedback from the teacher (competence-undermining), and communication issues with peers (relatedness-undermining). Chen and Jang (2010) used structural equation modeling to test a model for online learner motivation based on SDT. While they found support for the association of contextual support, satisfaction of the three basic needs and student motivation, self-reported motivation failed to predict learning outcomes. However, in a similar approach, Hsu et al. (2019) showed that satisfying the basic needs enhances self-regulated motivation, which is associated with higher perceived knowledge transfer and increased achievement of course objectives. Various studies showed that self-reported student motivation is positively associated with the quantity as well as quality of learning behavior in online teaching and learning settings, such as actively posting messages to an online learning platform (Xie et al., 2006; Hartnett, 2012). Xie et al. (2006) also found that student motivation is associated with teacher behavior, as for example, participation, guidance, and feedback.

The present study investigates how synchronous and asynchronous settings of teaching and learning during the 2020 lockdown affected student learning experience, including learning motivation, but also general satisfaction, learning behavior, and reported learning outcomes. The presented prior research on synchronous and asynchronous online learning stressed potentials and challenges of either setting, leading us to a partly explorative approach in this research to be able to provide a description of how synchronous and asynchronous teaching and learning settings in ERT were characterized by students and teachers regarding the applied teaching methods. A potential distinguishing factor between synchronous and asynchronous teaching and learning is how they facilitate social interaction between agents, why we chose to explore whether the settings differed in teaching methods and whether prerequisites for students engagement as proposed by SDT, (Ryan and Deci, 2000) are met differently between settings. Summarizing the above-mentioned studies on factors influencing online learning, we can classify them mainly in three groups – (1) learner-related variables (i.e., satisfaction, needs, and skills) (2) learning environment-related variables (i.e., synchronicity and potential for interactivity of online courses), and (3) teacher-related variables (i.e., applied teaching methods and teaching practices). Overall, we assume that a greater fulfillment of SDT needs should be associated with as more positive learning experience, as for example, a higher satisfaction with online learning and a higher reported support of SDT needs. Also, we assume that students who are more likely to accept online tools as useful and easy to use experience online learning during the pandemic as more positive.

Therefore, the following research questions frame our study:

Q1 a How are synchronous and asynchronous teaching and learning settings characterized by students and teachers regarding the applied teaching methods?

Q1 b Based on the proposed classification of methods regarding their potential to facilitate social interaction: What types of interaction are promoted in synchronous and asynchronous teaching and learning settings as reported by students and teachers?

Q2 Do students who experienced mostly asynchronous online teaching and learning report different overall evaluations of the online semester, fulfillment of basic psychological needs (SDT) as well as different learning gains compared to their peers who experienced mostly synchronous online teaching and do the teachers’ views validate students’ evaluations?

Q3 Is a more positive learning experience (overall evaluations of the online semester, self-reported learning gain) associated with

a) greater fulfillment of students’ basic psychological needs proposed by SDT?

b) greater acceptance of online tools?

## Materials and Methods

### Sample

The study reports data from both a student and a teacher online survey from a large German public university. The surveys were initiated by the university’s department of teaching and quality assurance in collaboration with representatives of other departments associated with teaching and learning. About 46,000 students are enrolled at the university, which employs about 3,500 research and teaching faculty. For the surveys, a randomly selected 50% percent of the student body and the teaching faculty were contacted, while making sure that teachers and students from all faculties received invitations. The other 50% of students and faculty were invited to participate in another survey focusing on examinations during COVID-19, the results of which are not part of the present paper. A total of 3,056 students completed the survey (return rate=15%, female=65.8%) as well as 396 teaching faculty (return rate=33%, female=39.1%). Table 1 contains further information about the student and teacher samples, including disciplinary clusters and students’ expected degrees. Both groups showed representativeness for the disciplines involved. On average, participating students were enrolled in their 4.9th semester (*SD*=3.34), and teachers reported an average number of 20.01 semesters (*SD*=17.43) of teaching experience. Note that students and teachers represent independent samples within the university and are not matched.

**Table 1:** Student and teacher samples by disciplinary cluster and expected degree (for students).

	Students		Teachers	
	*n*	%	*n*	%
**Disciplinary cluster**				
Humanities	786	25.7	136	34.3
Social sciences	882	28.9	126	31.8
Natural sciences	768	25.1	120	30.3
Teacher education	548	17.9	–^a^	–
Other (interdisciplinary, “I do not know,” n.s.)	72	2.4	14 (n.s.)	3.5
**Expected degree**				
BA	1,374	45		
MA	481	15.7		
State examination teacher	548	17.8		
State examination other	613	20.1		
Other (e.g., Magister, n.s.)	40	1.4		

a *All faculty members in teacher education are associated through their disciplinary faculty and listed thereunder*.

### Context of the Study

The surveys aimed to provide the university with a comprehensive feedback from students and teaching faculty on their experiences with the first online study term during the 2020 lockdown in Germany. This paper mainly reports select results from the student survey, but also refers to additional variables from the teacher survey to add a complementary perspective.

### Material

The student and the teacher surveys were carried out in German and were administered using EvaSys 7.0 software. The participation was voluntary and not linked to any credit. After providing their informed consent, participants anonymously answered the survey questions. Data were collected after the lecture period of the summer term; the survey was online from August until mid of September in 2020. All data were handled confidentially and securely on EvaSys and archived on a password-protected server. Due to the overall length of the surveys, all applied scales had to be shortened and were also adapted to fit the context of the study; other variables were measured through single items only. This article focuses on a number of selected variables that will be explained in further detail in the following.

### Student Survey

The student survey was designed to cover students’ views on the first online semester during the 2020 pandemic. It comprised background variables as well as evaluations of their study experience.

#### Teaching and Learning Methods

Students were asked to rate the frequency (1=*never* to 4=*very frequent*) of 11 different teaching and learning methods across all their courses. Teaching and learning methods were identified based on Alqurashi (2019) and included synchronous and asynchronous activities as well as methods that could be used in either setting (see Table 2). Following approaches differentiating learning activities in accordance with interaction types (Anderson, 2003; Chi, 2009), we propose a classification aimed to classify teaching and learning methods regarding their potential to facilitate social interaction (comprising learner-learner and learner-teacher interaction, see Table 2).

**Table 2:** Classification of teaching and learning methods classified regarding their synchronicity and potential to facilitate social interaction.

Synchronicity/ delivery form	Teaching and learning methods	Potential to facilitate social interaction	
		Lower	Higher
Synchronous	discussions *via* chat tools or videoconferencing/breakout rooms		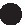
	practical work/labs		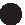
	group work		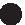
	(online) office hours		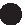
	lectures or student presentations *via* videoconferencing	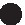	
Asynchronous	discussions *via* forums		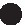
	self-tests or self-assessments *via* LMS	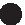	
	recorded lectures or student presentations	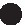	
Both synchronous and asynchronous	teacher feedback to students		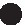
	peer feedback		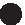
	student feedback to teacher		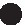

#### Individual Assessment of the Study Term

In single items, students were asked to evaluate their overall *satisfaction with the online term* (1=*strongly disagree* to 6=*strongly agree*), whether they experienced – in comparison with traditional teaching – *additional strains* (1=*strongly disagree* to 6=*strongly agree*) and additional workload through the online teaching. Students were also asked in which ratio they experienced *synchronous teaching and learning* across all their courses (1=*all synchronous* to 5=*all asynchronous*). Following the operationalization by Murphy et al. (2011), synchronous online teaching was understood as a temporally dependent arrangement between students and teachers, defined as weekly courses with fixed timeslots, whereas asynchronous teaching was defined by the absence of fixed weekly time slots, that is, temporally independent.

#### Self-Determination

To assess the perceived fulfillment of the base psychological needs proposed by SDT, we applied a questionnaire by Rösler et al. (2016). In three subscales, *autonomy support, competence support, and relatedness* were assessed by three items each:

- Autonomy support: (1) *I was able to complete assigned tasks my way*. (2) *I was able to manage time in my studies myself*. (3) *I had the opportunity to engage with contents I found interesting more intense*.
- Competence support (1) *I received clear and detailed feedback on my learning results*. (2) *I was provided with distinct information on how to improve*. (3*) When there were difficulties, I was able to get support at any given time*.
- Relatedness (1) *Overall, I experienced a feeling of belonging in my virtual courses*. (2) *The atmosphere amongst students was friendly and relaxed*. (3) *I felt comfortable amongst my fellow students*.

All items were answered on a 6-point rating scale (1=*does not apply* to 6=*fully applies*; autonomy support: *α* =0.75; competence support: *α* =0.86; relatedness: *α*=0.81).

#### Learning Gain

Students were asked to rate their *overall gain* in five distinct learning areas: content-related skills, method-related skills, digital skills, content interest, and autonomous learning (1=*very little* to 6=*considerably*).

#### Learning With Digital Tools

To assess the quality of learning with digital tools, we included a single item: Whether the constant availability of learning material led students to procrastinate (1=*does not apply* to 6=*fully applies)*. We also included a shortened version of the *learning content interaction* subscale from a questionnaire by Alqurashi (2019) to measure how students judged their learning with online material (learner-content interaction, LCI). The three items were rated from 1=*does not apply* to 6=*fully applies* (*α*=0.91): (1) O*nline course materials helped me to understand better the class content*. (2) *Online course materials stimulated my interest for this course*. (3) *Online course materials helped relate my personal experience to new concepts or new knowledge*. Referring to the Technology Acceptance model (Davis, 1989; Davis et al., 1989), we assessed the *perceived ease of use* (PEOU) as well as the *perceived usefulness* (PU) of online tools in teaching by each two items, that were answered on a 6-point rating scale (1=*does not apply* to 6=*fully applies* (PEOU: *α* =0.82; PU: *α* =0.85).

- Perceived ease of use: (1) *I find the online tools in teaching easy to use*. (2) *I find online tools in teaching to be flexible to interact with*.
- Perceived usefulness: (1) *Using online tools in teaching makes my learning more effective*. (2) *I find the online tools in teaching useful in structuring my learning*.

#### Teacher Survey

From the comprehensive teacher survey, we focus on the following selection of single items:

*Evaluation of own teaching*. Teachers were asked to rate their *overall satisfaction* with the online term (1=*strongly disagree* to 6=*strongly agree*) and to compare the *effort to prepare and perform teaching* with their usual experience (1=*far less* to 6=*far more*). Furthermore, they were asked to state whether their *digital competences* enhanced during the online semester (1=*very little* to 6=*considerably*). As with the students, teachers were asked to rate whether they taught more synchronously or asynchronously on a 5-point rating scale (1=*all synchronous* to 5=*all asynchronous*) as well to rate the frequency (1=*never* to 4=*very frequent*) of teaching and learning methods across all their courses (see Table 2).

*Evaluation of student variables*. Teachers were asked to rate whether students seemed to be more burdened in this semester than they usually are, whether students seemed to be overwhelmed by the number of digital tools, and whether the teacher thought that most of their students had problems in organizing their own learning at home. All three items were answered on a 6-point rating scale from 1=*does not apply* to 6=*fully applies*.

### Analyses

Based on the nature of our research questions, we included descriptive analyses (Q1a and Q1b), as well as analyses of group differences (Q1a, Q1b, and Q2), and the evaluation of associations between variables (Q3). To address possible group differences, we computed univariate ANOVAs. For associations between variables, we applied two-sided Pearson’s correlations. Data were analyzed using SPSS (Version 26).

## Results

**Figure 1:**
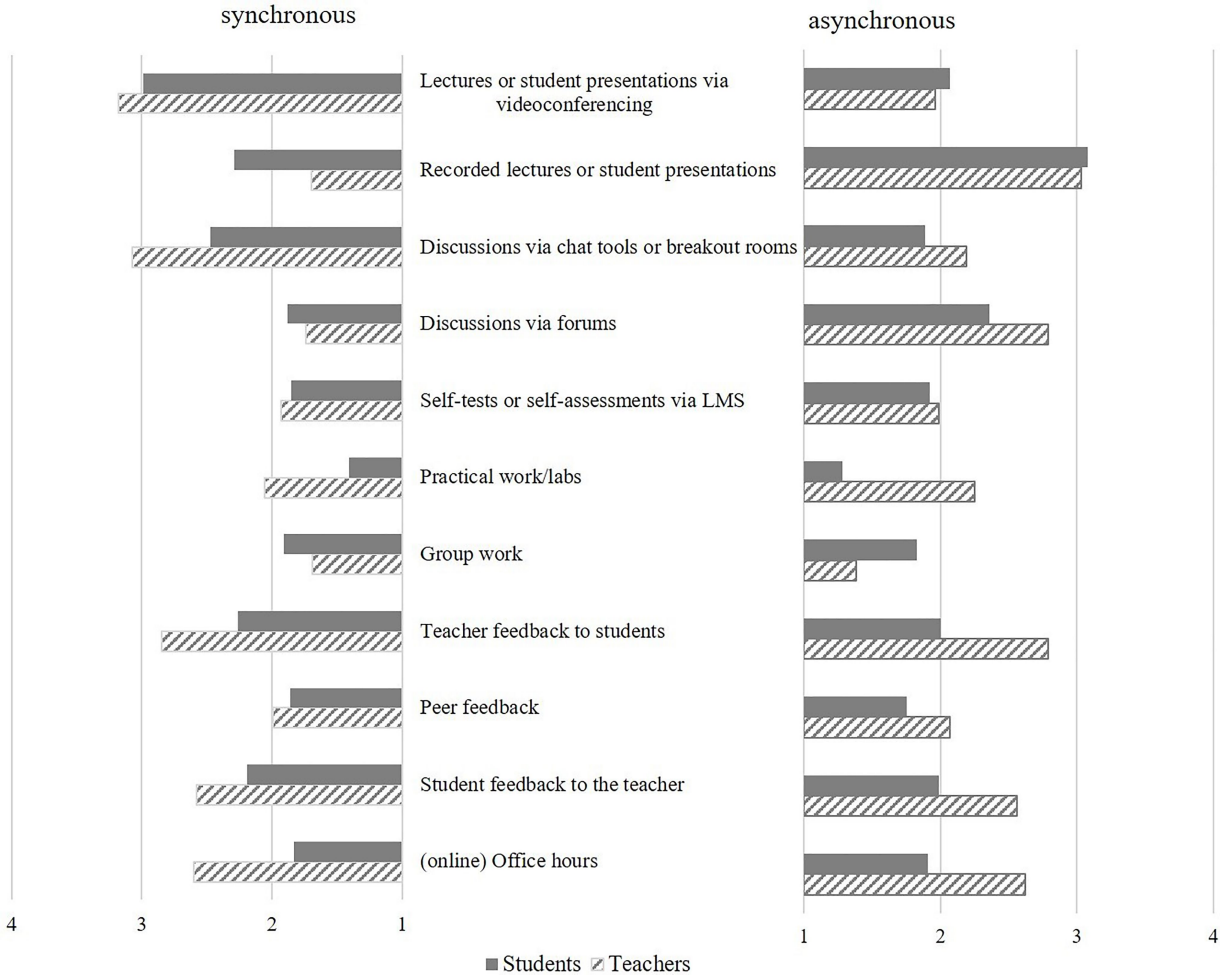
Reported mean frequencies of teaching and learning methods (1=*never* to 4=*very frequent*) in synchronous vs. asynchronous settings by students and teachers.

In preparation to further analyses, we dichotomized *synchronicity of teaching* for students and teachers to enable a comparison of extreme groups. For both samples, we merged the lower two (= mostly synchronous, students; *n*=1,020; 33.4%; teachers: *n*=149; 37.6%) and the upper two values (= mostly asynchronous, students: *n*=825; 27%; teachers: *n*=130; 32.9%) while omitting the middle category (= “a bit of both,” students: *n*=999; 32.7%; teachers: *n*=100; 25.3%)

(1a) To answer the research question how synchronous and asynchronous teaching and learning settings are characterized by students and teachers regarding the applied teaching methods, we first viewed the reported frequencies as a function of the two teaching and learning settings (see Figure 1, for the exact descriptive statistics, see Tables 3, 4). The descriptive results show that lectures and presentations were by far the most common method – videotaped for the mostly asynchronous groups and live *via* videoconferencing for the mostly synchronous groups. Unsurprisingly, practical work was reported as least frequent in all groups. We followed up with a more detailed analysis of the descriptive results.

**Table 3:** Descriptive results for groups and group comparisons of student perceptions of teaching and learning methods.

	Mostly synchronous			Mostly asynchronous			
Measure	*n*	*M*	*SD*	*n*	*M*	*SD*	ANOVA
Lectures or student presentations *via* videoconferencing	999	2.99	0.88	811	2.07	0.75	*F*(1, 1,808)=558.25, *p* =0.00, *η*^2^ =0.24
Recorded lectures or student presentations	978	2.29	0.97	807	3.08	0.97	*F*(1, 1,783)=292.57, *p* =0.00, *η*^2^ =0.14
Discussions *via* chat tools or breakout rooms	978	2.47	0.96	807	1.89	0.73	*F*(1, 1,783)=198.84, *p* =0.00, *η*^2^ =0.10
Discussions *via* forums	973	1.88	0.85	807	2.36	0.98	*F*(1, 1,778)=123.74, *p* =0.00, *η*^2^ =0.07
Self-tests or self-assessments *via* LMS	981	1.85	0.89	812	1.92	0.90	*F*(1, 1,791)=2.30, *p* =0.09, *η*^2^ =0.00
Practical work/labs	966	1.41	0.81	801	1.28	0.62	*F*(1, 1,765)=14.22, *p* =0.00, *η*^2^ =0.01
Group work	978	1.91	0.96	810	1.83	0.93	*F*(1, 1,786)=3.26, *p* =0.07, *η*^2^ =0.00
Teacher feedback to students	978	2.26	0.86	804	2.00	0.76	*F*(1, 1,780)=45.73, *p* =0.00, *η*^2^ =0.03
Peer feedback	986	1.86	0.88	801	1.75	0.82	*F*(1, 1,785)=8.28, *p* =0.00, *η*^2^ =0.01
Student feedback to the teacher	975	2.19	0.77	806	1.99	0.74	*F*(1,1,779)=31.64, *p* =0.00, *η*^2^ =0.02
(online) Office hours	955	1.83	0.85	788	1.91	0.82	*F*(1, 1,741)=3.16, *p* =0.08, *η*^2^ =0.00

**Table 4:** Descriptive results for groups and group comparisons of teacher perceptions of teaching and learning methods.

	Mostly synchronous			Mostly asynchronous			
Measure	*n*	*M*	*SD*	*n*	*M*	*SD*	ANOVA
Lectures or student presentations *via* videoconferencing	147	3.18	1.05	75	1.96	0.83	*F*(2, 269)=89.34, *p* =0.00, *η*^2^ =0.40
Recorded lectures or student presentations	146	1.70	0.95	75	3.03	1.10	*F*(2, 272)=47.81, *p* =0.00, *η*^2^ =0.26
Discussions *via* chat tools or breakout rooms	146	3.07	1.02	74	2.19	0.84	*F*(2, 267)=65.92, *p* =0.00, *η*^2^ =0.33
Discussions *via* forums	145	1.74	0.91	75	2.79	1.00	*F*(2, 269)=34.33, *p* =0.00, *η*^2^ =0.20
Self-tests or self-assessments *via* LMS	146	1.93	0.98	75	1.99	1.05	*F*(2, 271)=0.79, *p* =0.46, *η*^2^ =0.01
Practical work/labs	143	2.06	1.07	75	2.25	1.02	*F*(2, 268)=1.34, *p* =0.26, *η*^2^ =0.01
Group work	141	1.69	1.03	73	1.38	0.86	*F*(2, 262)=2.66, *p* =0.07, *η*^2^ =0.02
Teacher feedback to students	147	2.85	0.92	72	2.79	0.90	*F*(2, 269)=0.13, *p* =0.88, *η*^2^ =0.00
Peer feedback	145	1.99	1.00	74	2.07	1.04	*F*(2, 264)=0.13, *p* =0.88, *η*^2^ =0.00
Student feedback to the teacher	141	2.58	0.94	73	2.56	0.76	*F*(2, 263)=2.41, *p* =0.09, *η*^2^ =0.02
(online) Office hours	145	2.60	1.02	73	2.62	0.86	*F*(2, 268)=6.28, *p* =0.00 *η*^2^ =0.05

We conducted two univariate ANOVAs to test the assumption that the frequency of reported teaching and learning methods is dependent on the synchronicity of courses participated in (for students) or conducted (for teachers). The results revealed that students in the mostly asynchronous group reported significantly more recorded lectures or student presentations, as well as more discussions *via* online forums (LMS), with both methods being an integral part of the concept of asynchronous settings (see Table 3). Students in the mostly synchronous group reported significantly more lectures or student presentations *via* videoconferencing as well as more discussions *via* chat tools or breakout rooms, with both methods being an integral part of the concept of synchronous settings. As expected, students experiencing mostly synchronous settings also reported significantly more practical or lab work. They also reported higher frequencies for all the three forms of feedback activities (peer feedback, teacher feedback, and student feedback to the teacher) which are not conceptually tied to a specific setting. No significant differences could be found in reported frequencies of group work, self-assessments, and (online) office hours between synchronous and asynchronous groups.

In addition, teachers in the mostly asynchronous group reported high frequencies of recorded lectures or student presentations and organizing discussions *via* forum (LMS; Table 4). Additionally, the offer of online office hours was significantly higher in the mostly asynchronous group compared to the mostly synchronous. Similar to the student perspective, lecturing and organizing discussions *via* videoconferencing were also perceived by teachers in the mostly synchronous group as significantly more prevalent. No significant differences from the teachers’ perspective could be found for the reported frequencies of group work, self-assessments, and practical work/laboratories as well as for all three types of feedback (peer feedback, teacher feedback, and student feedback to the teacher) between synchronous and asynchronous groups.

Thus, students and teachers perceived the teaching and learning methods in synchronous and asynchronous settings differently: Teachers perceived fewer difference between teaching and learning methods in synchronous and asynchronous settings compared to students, especially in relation to feedback activities, which students reported as more frequent in synchronous settings. Another difference relates to (online) office hours that teachers offer more frequently when they teach more asynchronously compared to the mostly synchronous group. Here, students reported no difference

(1b) To answer the research question concerning prevailing types of interaction (lower vs. higher potential to facilitate social interaction) in synchronous and asynchronous teaching and learning settings, we qualitatively analyzed the reported teaching and learning methods, based on the proposed classification of their potential to facilitate social interaction displayed in Table 2. In summary, students in the mostly synchronous group experienced more teaching and learning activities with higher potential to support social (practical or lab work as well as the three types of feedback activities) as opposed to methods with lower potential to support social interaction (e.g., lectures *via* videoconferencing). In contrast, students in the mostly asynchronous group reported more methods with lower potential to facilitate social interaction (e.g., tests and recorded lectures) as opposed to methods with higher potential to support social interaction (e.g., forums and feedback activities). At the same time, teachers perceived teaching and learning methods in both learning environments as balanced in facilitating all types of interaction

(2) Regarding the second research question, student variables on the individual learning experience, SDT, and the reported learning gain were compared for group differences. For an overview, descriptive results of student variables are displayed in Table 5 across all students together with their intercorrelations. Almost all of the intercorrelations are significant indicating a likely overall factor behind the student ratings.

**Table 5:** Descriptive results and intercorrelations of student variables included.

Variable		*n*	*M*	*SD*	1	2	3	4	5	6	7	8	9	10	11	12	13	14	15	16	17
1.	Satisfaction overall	2,937	3.79	1.41	–																
2.	Additional strains	2,922	4.14	1.69	−0.54																
3.	Additional workload	2,725	4.09	1.45	−0.36	0.49															
4.	Procrastination	2,746	3.46	1.66	−0.24	0.28	0.12														
5.	LCI	2,828	3.69	1.27	0.66	−0.46	−0.32	−0.24													
6.	Autonomy support	2,848	4.23	1.06	0.56	−0.49	−0.36	−0.25	0.60												
7.	Competence support	2,816	3.08	1.25	0.48	−0.38	−0.26	−0.15	0.49	0.46											
8.	Relatedness	2,805	3.84	1.25	0.52	−0.39	−0.26	−0.23	0.52	0.49	0.53										
9.	Content skills	2,881	3.87	1.31	0.56	−0.38	−0.24	−0.27	0.64	0.57	0.41	0.47									
10.	Method skills	2,827	3.48	1.35	0.51	−0.32	−0.21	−0.21	0.56	0.50	0.44	0.45	0.70								
11.	Vocational skills	2,746	2.75	1.45	0.36	−0.26	−0.18	−0.15	0.41	0.35	0.34	0.34	0.46	0.50							
12.	Social skills	2,840	2.24	1.28	0.33	−0.25	−0.14	−0.13	0.37	0.30	0.35	0.43	0.35	0.42	0.49						
13.	Digital skills	2,883	3.91	1.32	0.37	−0.21	−0.12	−0.09	0.39	0.36	0.30	0.36	0.38	0.40	0.33	0.38					
14.	Interest	2,826	3.71	1.34	0.54	−0.38	−0.27	−0.22	0.63	0.56	0.43	0.48	0.72	0.59	0.46	0.39	0.45				
15.	Autonomous learning	2,872	4.45	1.36	0.41	−0.27	−0.13	−0.24	0.45	0.47	0.27	0.36	0.53	0.48	0.34	0.31	0.48	0.56			
16.	Perceived ease of use	2,860	4.39	0.99	0.43	−0.30	−0.22	−0.15	0.40	0.42	0.30	0.38	0.36	0.31	0.21	0.17	0.24	0.33	0.26		
17.	Perceived usefulness	2,843	3.53	1.20	0.61	−0.47	−0.33	−0.28	0.66	0.59	0.48	0.53	0.56	0.49	0.40	0.36	0.37	0.55	0.46	0.57	–

*LCI=Learner-content Interaction. All correlations (Pearson’s r) are significant (p<0.001)*

Table 6 displays the descriptive results for the two student groups with primarily synchronous and asynchronous teaching. Descriptive statistics for the selected teacher variables can be found in Table 7. The results suggest an overall medium to high satisfaction in both groups but also relatively high absolute ratings for additional strains and additional workload. To test for significant group differences, we conducted a univariate ANOVA for the student variables (see Table 6 for a summary of results) as well as one for the teacher variables (see Table 7). While the focus lied on students’ results, we report corresponding results from the teacher survey to add another perspective wherever possible. Students in mainly synchronous settings were significantly more satisfied with teaching across all their courses. It may be interesting to add that teachers mostly involved in synchronous settings were themselves more satisfied with the online term than those teaching in mostly asynchronous settings were. Students in mostly asynchronous settings reported a higher additional workload compared to teaching in face-to-face settings than did their peers in the synchronous group. We also found a significant difference between the two groups of students in terms of the perceived additional strains during the online term, even though the question was not directly related to teaching scenarios. Students in the asynchronous group report higher scores, which is also confirmed by the corresponding result from the teacher survey (see Table 7). For the SDT-related variables, we find significant differences between the two groups with higher values for competence support and relatedness in the synchronous group and higher values for autonomy support in the asynchronous group. The group with mostly synchronous teaching also reports significantly higher ratings in gaining procedural and social skills, as well as in their interest in the disciplinary content. Students with mostly asynchronous teaching report greater gains in self-directed learning compared to the other group. No differences were found in students’ learning gains regarding content skills, vocational skills, and digital skills. About half of teachers reported that most of their students had problems with self-organizing their learning at home

**Table 6:** Descriptive results for groups and group comparisons of student variables.

	Mostly synchronous			Mostly asynchronous			
Measure	*n*	*M*	*SD*	*n*	*M*	*SD*	ANOVA
Satisfaction	1,001	4.02	1.39	816	3.73	1.40	*F*(1, 1,815)=20.25, *p* =0.00, *η*^2^ =0.01
Additional strains	978	3.92	1.73	795	4.17	1.67	*F*(1, 1,771)=9.59, *p* =0.00, *η*^2^ =0.01
Additional workload	956	3.76	1.49	788	4.20	1.38	*F*(1, 1,742)=41.15, *p* =0.00, *η*^2^ =0.02
Procrastination	923	3.41	1.65	802	3.47	1.73	*F*(1, 1,723)=0.62, *p* =0.43, *η*^2^ =0.00
LCI	974	3.74	1.31	811	3.73	1.27	F(1, 1,783)=0.06, *p* =0.81, *η*^2^ =0.00
**SDT**							
Autonomy support	994	4.27	1.11	809	4.38	1.02	*F*(1, 1,801)=4.64, *p* =0.03, *η*^2^ =0.00
Competence support	980	3.33	1.31	798	2.90	1.21	*F*(1, 1,776)=51.28, *p* =0.00, *η*^2^ =0.03
Relatedness	990	4.02	1.28	786	3.72	1.29	*F*(1, 1,774)=23.88, *p* =0.00, *η*^2^ =0.01
**Learning gain**							
Content skills	977	3.95	1.31	789	3.95	1.26	*F*(1, 1,764)=0.01, *p* =0.93, *η*^2^ =0.00
Method skills	952	3.61	1.35	777	3.48	1.35	*F*(1, 1,727)=4.08, *p* =0.04, *η*^2^ =0.00
Vocational skills	922	2.79	1.47	745	2.75	1.43	*F*(1, 1,665)=0.36, *p* =0.55, *η*^2^ =0.00
Social skills	956	2.35	1.34	774	2.11	1.19	*F*(1, 1,728)=15.67, *p* =0.00, *η*^2^ =0.01
Digital skills	976	3.99	1.36	783	3.92	1.29	*F*(1, 1,757)=1.30, *p* =0.25, *η*^2^ =0.00
Interest	954	3.84	1.37	775	3.69	1.31	*F*(1, 1,727)=5.53, *p* =0.02, *η*^2^ =0.00
Self-directed learning	969	4.41	1.39	787	4.57	1.30	*F*(1, 1,754)=5.92, *p* =0.02, *η*^2^ =0.00

*LCI=Learner-content interaction. All variables were rated from 1 to 6, with 6 indicating higher values*.

**Table 7:** Descriptive results for groups and group comparisons of teacher variables.

	All			Mostly synchronous			Mostly asynchronous			
	*n*	*M*	*SD*	*n*	*M*	*SD*	*n*	*M*	*SD*	ANOVA
**Evaluation of own teaching**										
Overall satisfaction	274	4.28	1.07	147	4.47	1.04	73	4.17	1.03	*F*(2, 271)=6.61, *p* =0.00, *η*^2^ =0.04
Additional effort	272	4.79	0.92	146	4.66	0.92	72	5.00	0.85	*F*(2, 269)=3.56, *p* =0.03, *η*^2^ =0.02
Gain digital competence	246	4.36	1.05	130	4.43	1.08	71	4.46	1.02	*F*(2,243)=2.98, *p* =0.05, *η*^2^ =0.02
**Evaluation of students**										
Additional strains	228	4.27	1.27	125	4.06	1.30	68	4.51	1.21	*F*(2, 225)=3.79, *p* =0.02, *η*^2^ =0.03
Overburdening by number of tools	246	3.12	1.34	134	2.80	1.27	73	3.50	1.25	*F*(2, 243)=8.56, *p* =0.00, *η*^2^ =0.06
Problems in organizing learning at home	222	3.48	1.23	119	3.31	1.31	67	3.76	1.03	*F*(2, 219)=2.79, *p* =0.06, *η*^2^ =0.02

*All variables were rated from 1 to 6, with 6 indicating higher values*.

(3) To answer Q3, we refer to the correlational data reported in Table 5. We were interested in whether higher values in SDT as well as in PEOU and PU are associated with a more positive learning experience and can therefore act as protective factors for students. For these analyses, we refer to the complete set of students’ data. Following the assumptions of SDT, we expected that students whose basic psychological needs of autonomy, competence, and relatedness were more satisfied in the online semester also report greater overall satisfaction with the online semester as well as greater learning gains. Correlations between students’ overall satisfaction and the three basic needs range from 0.48 to 0.56 and were all significant, confirming our expectation. Correlations between the three basic needs and self-reported learning gains were also all positive and significant, ranging from 0.30 to 0.57, with the associations between the perceived support of autonomy and the different kinds of self-reported learning being the strongest. All three basic needs were also significantly negative associated with perceived additional strains during the online semester as well as with procrastination behavior. We further assumed that high technology acceptance should ease students learning experience in the online semester. Correlations between PEOU and student variables ranged from 0.17 to (−)0.43 and were all significant (*p*<0.001), with the highest coefficients for the association with overall satisfaction (*r*=0.43), LCI (*r*=0.40) and autonomy support (*r*=0.42). The perceived usefulness of online tools showed correlations between *r*=0.28 and *r*=0.66. All correlations were significant (*p*<0.001), and none of the directions was counterintuitive. Yet, we only found moderate to strong correlations. Students high in perceived usefulness judged their overall satisfaction with the online term positive as well (*r*=0.66) and reported less additional strain (*r*=−47). PU also positively correlates with higher perceived quality of learner-content interaction (LCI, *r*=0.66) as well with the three SDT needs (autonomy support: *r*=0.59; competence support: *r*=0.48; relatedness: *r*=0.53). Moderate positive correlations occurred also with reported learning gains for content skills (*r*=0.56), method skills (*r*=0.49), vocational skills (*r*=0.4), interest (*r*=0.55), and autonomous learning (*r*=0.46).

## Discussion

Through the work presented in this article, we aim to understand better, how university students and teachers experienced different settings of online teaching and learning during the online semester due to the COVID-19 lockdown. In particular, this study aims to comprehend the effects of mostly synchronous and mostly asynchronous teaching and learning settings on students and at providing insight into possible implications for future online teaching and learning in higher education. Based on the results of a university-wide survey, we analyzed whether synchronous and asynchronous teaching and learning settings were associated with different teaching methods as well as differences in various student variables.

### Discussion of Results

*Teaching and learning activities in synchronous and asynchronous setting involve less interaction inducing methods than input methods*.

The first research question explores which teaching methods were reported by students (and teachers) who experienced mostly synchronous or asynchronous online teaching and learning. Results show that considerable groups of students experienced teaching that was predominantly either synchronous or asynchronous. Only about one-third of students reported equal ratios of both settings. Even though a wide variety of methods was reported, results show that synchronous and asynchronous online courses were dominated by prepared inputs by students, teachers, or both, such as live presentations during video conferencing or previously recorded lectures or screencasts.

However, the frequency of the methods reported by students and teachers depended on the synchronicity of the courses. Unsurprisingly, students and teachers who studied or taught mostly asynchronous reported more methods that are conceptually tied to asynchronous settings (e.g., recorded lectures or student presentations and discussions *via* online forums) compared to students and teachers in mostly synchronous settings. Vice versa, students and teachers in mostly synchronous settings reported more synchronous methods (e.g., presentations *via* videoconferencing, discussions *via* chat tools or breakout rooms) compared to students and teachers in mostly asynchronous settings. These results were expected because methods such as recorded lectures are inevitably applied more often in asynchronous settings while videoconferencing can only be realized in synchronous settings. Nevertheless, these results may serve as confirmation that our segmentation of the sample into a *mostly synchronous group* and a *mostly asynchronous group* was admissible.

Our findings reveal discrepancies regarding student and teacher perceptions of the frequency of methods that facilitate interaction in synchronous and asynchronous settings.

It has to be noted that synchronous and asynchronous settings differ in principle regarding their potential to facilitate social interaction: Synchronous environments allow for teaching methods such as group work or video discussions, which inherently support social interaction of students as well as student-teacher interaction. In comparison, asynchronous environments are more content-oriented and teaching methods conceptually tied to asynchronous settings have a focus on facilitating student interaction with the learning materials. Asynchronous methods that facilitate social interaction such as discussions in online forums require more attention as well as a more thorough planning in order to support social interaction compared to for example discussions in video conferences. However, all three forms of feedback activities (peer feedback, teacher feedback to students, and student feedback to the teacher) can be realized in both synchronous and asynchronous settings. Yet, our data suggest that students in mostly synchronous settings experience more feedback compared to students in mostly asynchronous settings.

Interestingly, the students’ perception of feedback activities in synchronous and asynchronous settings in our study is not confirmed from the teachers’ perspective: Teachers reported to apply all three feedback activities (as well as group work and practical work/labs) equally in both asynchronous and synchronous settings. One likely explanation for this discrepancy is that teachers are just not aware that they allow for less feedback in asynchronous settings compared to synchronous settings. Maybe some of the feedback activities that take place in synchronous settings occur unintentionally without being deliberately planned by teachers. In any case, given the pivotal role of informative feedback in (not only) higher education learning in order to assure motivation and learning outcomes (Biggs and Tang, 2011; Hattie, 2011), this finding may suggest a disadvantage for students experiencing mostly asynchronous teaching and learning settings. Similar differences in teacher and student perceptions were found earlier regarding preferences for interaction-based and input-based settings by Struyven et al. (2008). The authors found that these preferences were able to influence students’ overall perceptions of learning environments as well as their learning strategies and their performance, while it is known that for learning success, input formats usually depend on both attention and interest from the students (Rapanta et al., 2020).

*Students in synchronous settings report a more positive learning experience as well as greater support of their basic psychological needs*.

The second research question compares the two groups’ learning experiences. We find satisfaction rates for synchronous settings to be higher, indicating that the social aspects of teaching and learning (e.g., feedback and interaction), which from the students’ perspective are more prevailing in synchronous settings, play an important role for student satisfaction. Regarding the support of the three basic psychological needs as described by SDT, our presumption is confirmed that students’ needs to feel competent as well as socially related cannot be taken for granted, especially for asynchronous settings. This study thereby contributes further empirical evidence for the appropriateness of applying the SDT to online teaching and learning in higher education. Future research that systematically varies teaching methods could provide further insight as well as intervention studies in which teachers are trained to apply the principles suggested by SDT in their teaching.

Regarding the students’ self-reported learning gains, synchronicity of the online setting seems to be of minor importance: While unsurprisingly, a majority of students reported improving their digital skills – as did more than 80% of the teachers – there was no difference between synchronous and asynchronous settings. Likewise, students self-reported learning gains did not significantly differ with regard to content-related skills and vocational skills. However, students who experienced mostly asynchronous teaching report greater gains in autonomous learning and smaller gains in social skills, both results being immediately plausible since asynchronous settings are characterized by high degrees of autonomy and fewer possibilities for social exchange. In contrast, students who mostly experienced synchronous teaching reported a greater increase in interest in the course content than students in asynchronous settings, suggesting that the content-related exchange with others supports the evolvement of interest for a certain topic. In addition, students in mostly synchronous settings reported higher gains in methodological skills. These results complement the findings by Nguyen (2021), who found that students prefer synchronous settings. While these results suggest a superiority of synchronous teaching and may be interpreted in such way that more video conferences are needed in higher education, one could also conclude that for the particular case of emergency remote teaching due to the COVID-19 pandemic, teachers had difficulties tapping the full potential of asynchronous teaching and learning arrangements. With more time for thorough course planning, teachers have the possibility to incorporate intelligent opportunities for both teacher-student and student–student interactions and collaboration into their online courses (Alqurashi, 2019). In this sense, results should be used to optimize both types of learning arrangements and allow for their purposeful use. Hrastinski (2010) suggests that synchronous communication may be used to foster personal participation and to allow convergence on meaning as well as provide task-related and social support, especially when applied in smaller group settings and for less complex tasks. Also, according to Daft and Lengel’s (1984) media richness theory, media Daft and Lengel (1984), mediums differ in their capability to transmit information with while face-to-face communication being the richest medium. Reflected knowledge of the different capabilities of different media should allow teachers to rationalize their choices to enhance their students’ learning.

*Overall, greater fulfillment of psychological needs and higher acceptance of online tools go along with a more positive learning experience*.

The third research question investigates whether higher SDT values were also associated with a more positive learning experience and whether greater technology acceptance also served as a protective factor for students in that sense. Indeed, we found that higher satisfaction scores regarding the three basic needs according to SDT correlated positively with overall satisfaction and negatively with the perception of additional strains and reported procrastination. The differences between synchronous and asynchronous settings stress the importance of the support of relatedness (see also Chiu, 2021), to make up for the disadvantages that go along with asynchronous settings. Similar to the results by Hsu et al. (2019), we also found that needs fulfillment were positively correlated with all of the facets of self-reported competence gain. Together with the results from our second research question, this indicates that the satisfaction of basic psychological needs enhances students’ learning experience comprising higher satisfaction, less procrastination, and greater learning gains. At the same time, students reported more support for their three basic needs in synchronous learning settings. Aside from synchronicity, we also found a positive correlation between autonomy support and the PEOU of technology. It could be argued that through this, also the interaction with online learning content could be eased, resulting in the experience of more autonomy support. With these results, this study contributes to the existing evidence for the application of SDT in online learning and it provides a good starting point for theoretical and practical implications. Even though SDT-related results in this study may suggest that synchronous settings outperform asynchronous settings, there are many good reasons why higher education should not completely abandon asynchronous teaching and learning. In the correlative results, we found strong associations between the perceived usefulness of given online tools and a positive learning experience, implying that teachers in general should allow their students to experience the usefulness of the chosen tools.

### Limitations

Several limitations of the current study should be noted. As many other studies on experiences with remote learning due to the pandemic, the results rely on data that are derived from a single German university; therefore, the results can only be generalized to a limited extent. However, the university is large and includes a wide variety of disciplines and study programs. Universities in Germany are equipped similarly when it comes to basic infrastructure and the challenges of the COVID-19 pandemic created a comparable interruption of regular teaching and learning for everyone. Therefore, we assume that results should be transferrable, at least for the German context. The relatively low response rate might also have resulted in a self-selection bias of students and teachers with regard to possible systematic differences to the non-responding groups. The representativeness for the faculties still is encouraging as well as the variance in variables’ scores. Also, the SDT describes the needs as universal across individuals (Deci and Ryan, 2000). From this point of view, the aggregation of data across courses and disciplines as well as grouping teachers and students according to the synchronicity of online learning can compensate the absence of matching between student and teacher samples on the course level. Another challenge is the quality of data, in regard to known problems of self-report measures, which are susceptible to memory distortions and do not equal actual performance (Schellings and van Hout-Wolters, 2011). And while, as mentioned by Pekrun (2020), self-reports can deliver data of high validity in investigating motivational, cognitive, or emotional aspects of learning but they should be enhanced by other data sources. Albeit the validity of the data was partially increased be integrating responses from teacher survey and student survey – allowing to some extent the cross verification of the findings from teacher and student perspectives, it would still be desirable in the sense of data triangulation for future research to integrate other sources of data related to online learning. These could include, for example, the frequency and real-time use of LMS, chats, or videoconferencing as well as the number of downloads of recorded lectures or podcasts. Another possibility of data triangulation could be a better integration of qualitative data in addition to quantitative data enabling stronger validation of results. As a further limitation, it should be mentioned that in student evaluations of teaching, high intercorrelations are well-known, indicating a central factor that influences a student’s evaluation of the lecturer (Shevlin et al., 2000). Still, self-reports provide an opportunity for insight into cognitive, motivational, and behavioral processes on a broad level that can help to detect systematic correlations. Another limitation is the instruments used for this study: Scales had to be shortened in order to be included in the comprehensive student and teacher survey. Some information had to be collected through single-item measures. However, the internal consistencies of scales applied were good or very good and therefore ensure a certain psychometric quality. A general challenge of one-shot studies is that they only have a correlational scope and do not allow causal relationships to be established even if the theoretical assumptions suggest them. And while we were able to harness data from both the student and teacher surveys, we are unable to link both data sources so we do not know whether potential differential effects are covered. All of these limitations connote future research strategies, where, for example, fewer courses are researched in more depth.

### Conclusion

Overall, our findings contribute to theory because they further indicate that the synchronous and asynchronous settings are no uniform environments but offer a variety of different options for teaching and learning. Also, our results offer evidence for an association between these settings and prerequisites for student engagement and indicators for satisfaction and learning behavior and perceived learning outcomes. Our research focused on teaching and learning during the 2020 lockdown, but even if the post-COVID classroom will differ from the *ad hoc* circumstances experienced during the first lockdown, the experience has produced a vast amount of insights into opportunities, potentials and risks of digitally organized learning (Aristovnik et al., 2020). These highly valuable first-hand experiences with online teaching and learning under real life conditions need to be integrated with existing findings from systematic research on online learning to help to refine future higher education online teaching and learning. However, it should be kept in mind that cultural differences might affect learning experience when interpreting findings that stem from specific national contexts (Chiu, 2021). We have found SDT to serve as a valuable model in interpreting results, and we would encourage further research to add to empirical evidence of SDT in higher education and specifically in online learning.

The universal necessity to engage with online learning for the majority of teachers and students was challenging, but further strengthened the topic not only for those with a specific interest in digital media. Besides the boost in digital skills for students and teachers (and most likely for universities as institutions as well), it has become even more obvious that teaching in higher education should support active learner-centered learning, especially for online settings. The purposeful and intentional use of technologies to allow for adaptive and fair learning opportunities in higher education is of ongoing and even growing importance. It is upon teachers to successfully implement online tools into their teaching and to develop teaching and learning arrangements with tools that serve a transparent purpose and also do not neglect student interactions with teachers, as well as with fellow students and with content. With asynchronous teaching formats in particular, we conclude that teachers need to put extra effort into providing sufficient opportunities for students to interact not only with the learning content but also with the teacher and their fellow students. Online settings of teaching and learning hold potential, not only for self-pacing studying, but also for flipped learning arrangements, adaptivity for individual needs, cooperative tasks like wikis or blogs and for automated assessments. All of this should be accompanied by continuous support, not only for technical issues but also for quality teaching and learning in online environments. Therefore, teachers need to be empowered to make the most of digital advances (OECD, 2020) while having enough room to autonomously make their own decisions and relate to others in this process (Moorhouse and Kohnke, 2021).

## Data Availability Statement

The datasets presented in this article are not readily available because they resulted from the extraordinary evaluation of online teaching at Goethe university which was developed by a joint group of researchers and officials; for further use of the dataset, consent of the group has to be obtained. Requests to access the datasets should be directed to the authors.

## Ethics Statement

The studies involving human participants were reviewed and approved by the data security officer of Goethe university Frankfurt. The patients/participants provided their written informed consent to participate in this study.

## Author Contributions

SF, JM, and SS contributed to conception and design of the study and wrote sections of the manuscript. SF and JM performed the statistical analysis. All authors contributed to manuscript revision, read, and approved the submitted version.

## Conflict of Interest

The authors declare that the research was conducted in the absence of any commercial or financial relationships that could be construed as a potential conflict of interest.

## Publisher’s Note

All claims expressed in this article are solely those of the authors and do not necessarily represent those of their affiliated organizations, or those of the publisher, the editors and the reviewers. Any product that may be evaluated in this article, or claim that may be made by its manufacturer, is not guaranteed or endorsed by the publisher.
